# Silver Attached Graphene-Based Aerogel Composite Phase Change Material and the Enhancement of Thermal Conductivity

**DOI:** 10.3390/ma13153271

**Published:** 2020-07-23

**Authors:** Liang Zhang, Zhongke Shi, Buning Zhang, Jinhui Huang

**Affiliations:** 1 School of Automation, Northwestern Polytechnical University, Xi’an 710129, China; zhangl@mail.nwpu.edu.cn; 2 Guangzhou Key Laboratory for Efficient Utilization of Agricultural Chemicals, Zhongkai University of Agriculture and Engineering, Guangzhou 510225, China; zhangbuning@zhku.edu.cn; 3 New Energy Materials Technology Development Center, Guangzhou Vocational and Technical University of Science and Technology, Guangzhou 510550, China; jinhui_nupw@mail.nwpu.edu.cn

**Keywords:** nanocomposite, thermal properties, energy storage

## Abstract

Phased energy storage technologies are highly advantageous and feasible for storing and utilizing clean renewable energy resources, for instance, solar energy and waste heat, and it is an effective method to improve energy efficiency and save energy. However, phase change energy storage has some problems, for example, low thermal conductivity and phase change leakage, which lead to limited application. In this paper, anisotropic graphene aerogels were prepared by ice crystal template method with high thermal conductivity of graphene, and silver was attached to the pore wall graphene sheets and the graphene sheet boundaries of the aerogels. The results show that anisotropic graphene aerogels were successfully prepared, and SEM and EDS indicate that up to 9.14 at % silver was successfully attached to the graphene sheets and boundaries. The anisotropic thermal conductivity of the PArGO phase change composites after adsorption of the paraffin is significant, with a maximum axial thermal conductivity of PArGO of 1.20 W/(mK) and radial thermal conductivity of 0.54 W/(mK), compared to the pure paraffin (0.26 W/(mK)) increased by 362% and 108%, respectively. The enthalpy of the composite has been reduced to 149.6 J/g due to the silver particles attached, but the thermal properties have been greatly improved. In experiments simulating real temperature changes, PArGO achieves phase transitions very fast, with a 74% improvement on thermal efficiency of storage and discharge over the pure paraffin.

## 1. Introduction

With the growing demand of energy consumption, the problem of shortages of common fossil fuels and the accompanying environmental pollution has become more serious. The need to find renewable and clean energy sources is becoming urgent. Thermal energy storage technology is one of the ideal solutions that offers clear economic advantages in terms of reducing energy wastage and costs. Moreover, it is also a clean and reusable technology. Phased energy storage technologies are highly advantageous and feasible for storing and utilizing clean renewable energy sources, such as solar energy and waste heat, and it is one of effective methods to improve energy efficiency and save energy. Phase change material (PCM) is a key factor in phase change energy storage technology.

There are many types of PCMs, including organic PCMs such as paraffin [1,2,3] and inorganic PCMs such as inorganic salt hydrates [4,5]. However, all of them have the problem of low thermal conductivity, which leads to low efficiency of phase change and the phenomenon of overcooling and overheating [6,7,8,9,10]. In addition, they are prone to leakage in the absence of containers and other encapsulation conditions [11,12]. Extensive research has been conducted to address these two key issues, and fins can be added and containers can be used to improve thermal conductivity and prevent leakage in some large applications. However, this is just an application at the technical level without changing the properties of the PCM itself, and it is unsuitable for some small or microscopic applications.

The emergence of graphene provides a good idea to solve both problems. Graphene has an ultra-high thermal conductivity, which is up to 5300 W/(mK) [13]. Thus, it can be used as a thermal conductive filler, greatly increasing the thermal conductivity of composite materials. In addition, in the preparation of aerogels, due to its high specific surface area and super capillary effect, it can adsorb PCMs very well, and thus have less or even no leakage. However, the large-scale preparation of graphene is still very difficult, and many still remain in the laboratory or research stage. The more economical preparation method is the oxidation-reduction method, in which graphene is first oxidized and dispersed, and then reduced to obtain a single layer or a few layers of graphene. However, the graphene prepared by this method has many defects such as doping, hole defects, and so on [14] which leads to a serious decrease in performance compared to the perfect graphene. The thermal conductivity of graphene is greatly affected by the way and degree of oxidation-reduction. However, it is widely used because of its simple process and low cost [15,16,17].

Many researchers have prepared graphene aerogels by oxidation-reduction method to adsorb PCMs and study their improvement effects on the thermal properties of composite phase change materials. It is found that graphene aerogel alone does not improve the thermal conductivity of phase change composites well, mainly because the random contact conduction of the graphene sheets increases the contact thermal resistance of the graphene sheets in the thermal conductivity pathway, while a large number of the graphene sheet boundaries also increase the boundary thermal resistance of the thermal conductivity pathway. Therefore, many researchers have proposed anisotropic graphene aerogels, in which the pore walls are relatively thick and the graphene sheets are regularly arranged so that the previously haphazard graphene sheets are tightly assembled into a single unit, thus reducing the contact thermal resistance and the boundary thermal resistance. Peng et al. used the ice crystal template approach to prepare anisotropic graphene aerogels, and the thermal conductivity was as high as 8.87 W/(mK) after adsorbing paraffin [7]. Li et al. deployed the method of solidifying the ordered liquid crystals of graphene oxide by the gaseous hydrogen chloride in situ, and then prepared anisotropic graphene aerogels. The thermal conductivity reaches 2.99 W/(mK) after absorbing paraffin [18]. Although anisotropic graphene aerogels can better decrease the thermal resistance and enhance the thermal conductivity, the thermal conductivity in the normal direction of the pore wall is still low, and the thermal resistance of the graphene sheet boundaries in contact with the PCM is still high.

Silver nanoparticles are of great importance owing to its high thermal conductivity of 429 W/(mK) at room temperature. They are expected to have good thermal properties preferably suitable for heat transfer applications [19,20] and phase change energy storage technologies [21,22]. Furthermore, silver is commonly considered as a safe material for human and animals. The addition of silver can not only improve the biocorrosion resistance of paraffin composite PCMs [23,24], but also reduce the aggregation of graphene during the thermal cycle [25].

The ice crystal template method, using cold source directed freezing of graphene wet gels, was used to prepare anisotropic graphene aerogels. Silver is attached to the graphene sheets and the boundaries, thereby greatly reducing the normal thermal conductivity of the pore wall and the contact thermal resistance between the graphene sheet boundaries and the PCM.

## 2. Experiments

### 2.1. Preparation of Graphene Oxide

A modified Hummers′ method [26] was used as following: an appropriate amount of concentrated sulfuric acid was added to a 250 mL reaction flask assembled in an ice water bath, and a solid mixture of expanded graphite (2 g) and sodium nitrate (1 g) was added under mechanical agitation, then potassium permanganate (6 g) was slowly added, stirred and mixed well, slowly raised temperature to 40 °C, and stirred for 4 h. Then the mixture was slowly diluted by adding 5 wt % H_2_SO_4_ solution on an ice bath below 5 °C after cooled to room temperature. During dilution, the temperature of the solution should not exceed 5 °C. Subsequently, H_2_O_2_ (30 wt %, 30 mL) was added, the solution turned bright yellow, stirred reaction for 1 h before centrifugation. Following the solid was centrifugal washed with 10 vol % HCl for several times, then washed with 2 vol % HCl. Finally, the precipitate was washed with distilled water several times and freeze-dried to obtain graphene oxide (GO).

### 2.2. Preparation of Anisotropic Graphene Aerogels and Composite PCM

Obtained GO (0.4 g) and 50 mL of water were added into the glass bottle in the reaction kettle, and then the solid–liquid mixture was placed in an ultrasonic bath with a cold-water circulating system. After 1 h, added concentrated ammonia (500 µL) and stirred evenly, and then the reaction kettle was placed into the oven for 3 h at 90 °C. Next, the reactor was taken out and put in deionized water for two days, during which the water was changed every eight hours, followed by 3 h of directional freezing at −45 °C, and then freeze-dried for two days. Finally, the reduced graphene oxide aerogel (rGO) was obtained after being microwaved under vacuum. The obtained rGO samples were immersed into the heated and melted paraffin (#52, Guangzhou Chemical Reagent Factory, Guangzhou, China) and absorbed by vacuum for 30 min, and then taken out and cooled naturally to obtain paraffin/rGO composite material, denoted as PrGO.

### 2.3. Preparation of Silver Attached Graphene Aerogels and Composite PCM

2% ammonia solution was added dropwise into 20 mL of 2 wt % AgNO_3_ ethanol (20%) solution slowly until a large amount of precipitation occurred. After a small amount of acetaldehyde was added in the solution and stirred well, the obtained rGO samples were immersed into this solution at 40 °C for a period of time to obtain a graphene wet gel with silver attached. Then the wet gel was freeze-dried to obtain a graphene aerogel with silver attached (Ag-rGO). The prepared aerogel was immersed in the heated and melted paraffin (#52, Guangzhou Chemical Reagent Factory, Guangzhou, China) and absorbed by vacuum for 30 min, and then taken out and cooled naturally to obtain paraffin/Ag-rGO composite material, denoted PArGO.

### 2.4. Performance and Structural Testing

Scanning electron microscopy (SEM) images were acquired on an EVO18 microscope (Carl Zeiss Microscopy GmbH, Jena, Germany). Aerogel samples were pasted on a conductive adhesive without spraying Au and directly observed, but the composite PCM was surface sprayed with gold (25 mA, 45 s) before observation.

X-ray energy dispersion (EDS) analysis was performed using chemical microanalysis INCA 350 (Oxford Instruments, Oxon, UK) for the elemental analysis after surface treatment of the samples (ethanol rinsed and vacuum dried).

A differential scanning calorimetry (DSC) instrument was used to analyze the thermal properties of the PCM-adsorbed composite material samples using Q2000 (TA Instruments, New Castle, DE, USA), with the following detection conditions: N_2_ protection, 50 mL/min, and a temperature rise rate of 10 °C/min.

The thermal conductivity of PCM composite material samples was measured using the transient hot wire method on a thermal conductivity meter TC3100 (XIATECH Electronics Co., Ltd., Xi’an, China).

## 3. Results and Analysis

### 3.1. Aerogel Morphology and Silver Attachment Analysis

**Figure 1:**
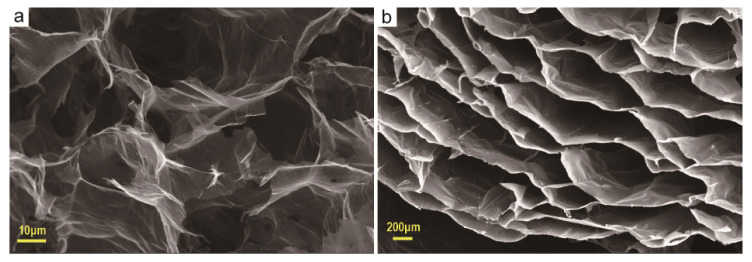
Electron micrographs of internal pore characteristics of graphene aerogels prepared by different methods (**a**) without directed freezing; (**b**) directed freezing.

**Figure 2:**
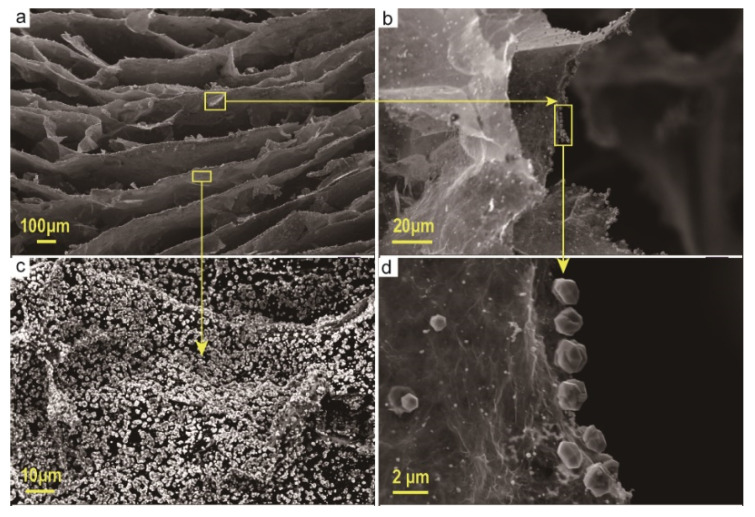
SEM image of the pore wall of the graphene aerogel attached silver (**a**) a cross-section of the aerogel; (**b**) the graphene sheets on the pore wall of the aerogel; (**c**) the silver particles attached to the aerogel pore wall; (**d**) the silver particles attached to the edge of the graphene sheet of the aerogel pore wall.

Anisotropic graphene aerogels were prepared by directional freezing. Figure 1a shows the aerogel prepared without directional freezing. It is obvious that the inner graphene sheets are randomly aligned in contact with each other and the pores also appear randomly, showing a haphazard and isotropic character. Figure 1b is an anisotropic graphene aerogel prepared by directional freezing. Due to the directional growth of ice crystals, the graphene sheets are crowded and aggregated into a graphene layer with a certain orientation, and the pores exhibit a certain directionality. Figure 2 is an electron micrograph of the aerogel after silver attachment. Figure 2a is a cross-sectional view of the aerogel. It can be seen from the figure that after the silver attachment process and freeze-drying again, the internal structure of the aerogel has not been destroyed and still retains a relatively stable anisotropic structure. Figure 2c shows that the graphene layer on its pore wall is attached with a large number of metallic silver particles. Figure 2b,d show that the boundaries of the graphene sheets are similarly adhered with a large number of metallic silver particles.

**Figure 3:**
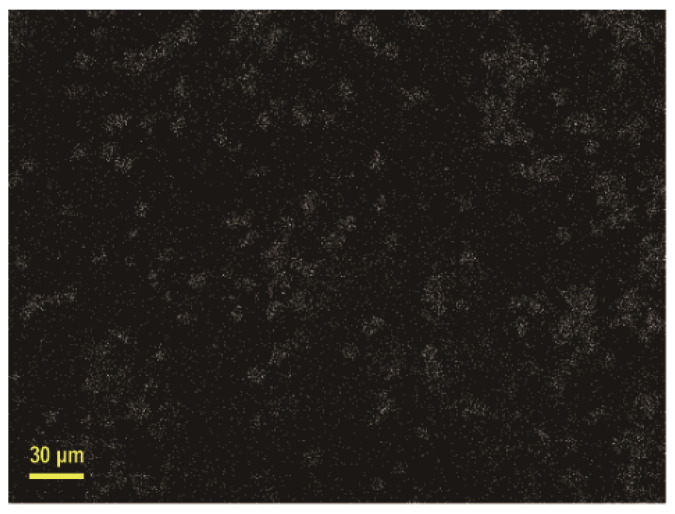
EDS surface scan images.

Further characterization by EDS verified the silver attachment on the graphene aerogel pore walls. From Figure 3, it can be seen that the silver elements with white dots are spread all over the graphene aerogel pore walls. The elemental spectrum shows that the silver element has a distinct spectral peak with a content as high as 9.14 at % (Table 1), indicating that the metallic silver was successfully attached to the graphene sheets.

### 3.2. Composite Phase Change Morphology and Thermal Properties Analysis

**Figure 4:**
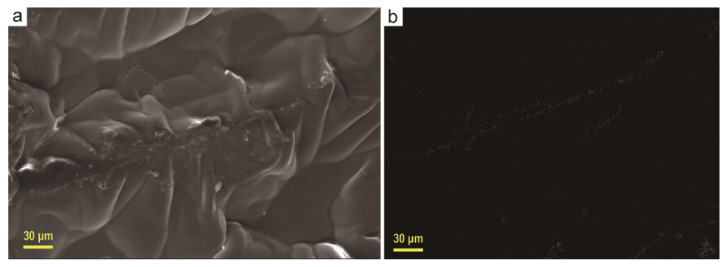
SEM image of PArGO (**a**) and EDS images of silver elemental plane scans (**b**).

After the prepared aerogel adsorbed paraffin, a composite phase change material was obtained, and its appearance and morphology were investigated as well as thermal properties. Figure 4a shows the apparent morphology of the aerogel after paraffin adsorption. It can be seen that there are a few small grooves in the middle of the two pore walls, which is caused by the volume shrinkage of the paraffin after the solidification. It is also clear from the figure that the paraffin is distributed along the pore wall and the pore wall traces are clearly visible. From the silver element scanned image of EDS in Figure 4b, it can be seen that its shape is consistent with the shape streak of the pore wall, which further indicates that a large amount of silver elements were attached to the graphene layers on the pore wall and did not completely fall off during the paraffin adsorption process.

**Figure 5:**
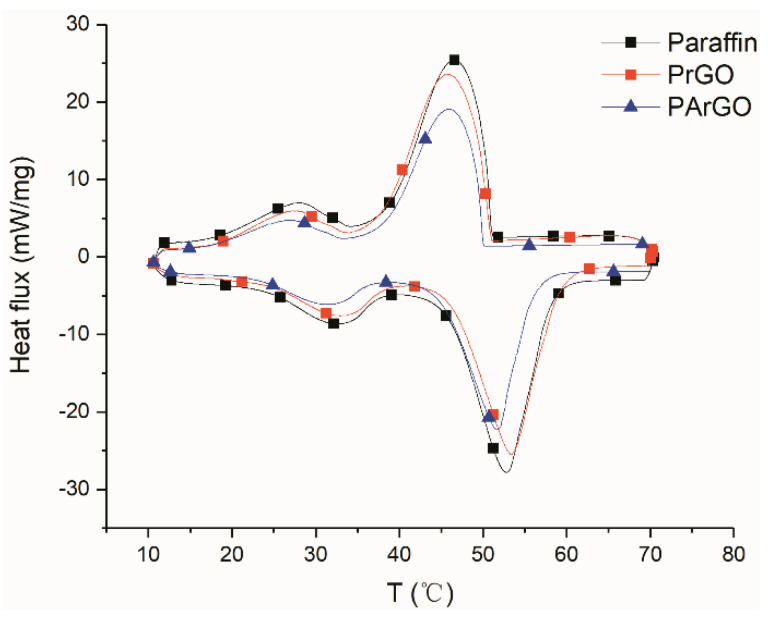
DSC curves for composite PCMs.

The thermal properties of the composite PCM were also tested. In Figure 5, the DSC test curves show that the phase transition temperature of PArGO is lower than that of paraffin and PrGO, which is due to its higher thermal conductivity with the attachment of silver, improving the thermal conductivity of the graphene sheets in the normal direction. At the same time, a large amount of silver is attached to the boundaries of the graphene sheets, which reduces the thermal resistance of heat transfer from the graphene sheets to the paraffin, thus increasing the thermal conductivity of the boundaries. This in turn leads to an increase in the thermal conductivity of the entire phase change composite, which reduces overheating. However, the phase transition temperature of PrGO was instead higher than that of paraffin. This is due to the fact that although the attachment of anisotropic graphene aerogels can improve the thermal conductivity of the composite PCM to some extent, there is a certain force between the graphene sheets and the paraffin [27,28]. This force slows down the movement of the paraffin molecules [29,30] and when the positive effect of increasing the thermal conductivity is lower than the force between the graphene sheets and the paraffin, a retardation of the phase transition behavior can be exhibited, resulting in an increase in the phase transition temperature compared with the pure paraffin. The graph also shows that the three materials have different peak heights for heat absorption and exertion. This is due to a decrease in the relative content of paraffin added to the aerogel, resulting in a decrease in the enthalpy, from 166.2 J/g of the pure paraffin to 149.6 J/g of PArGO.

**Figure 6:**
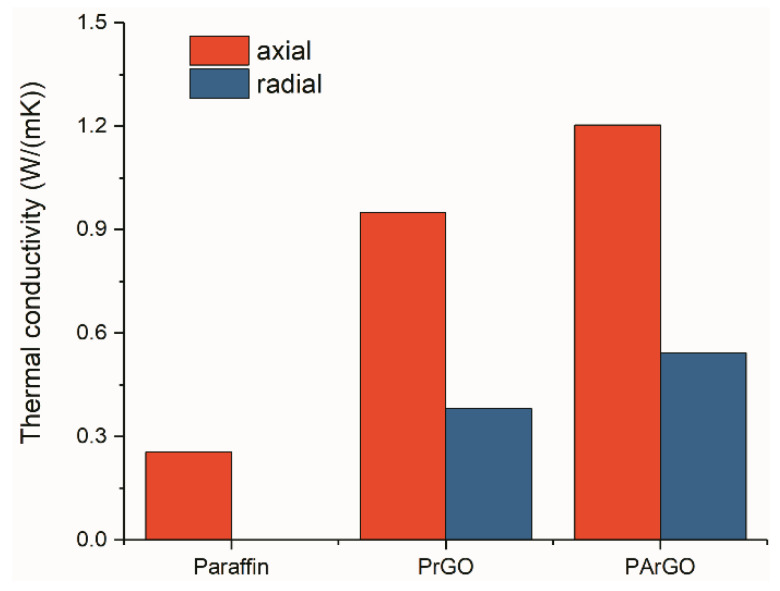
Thermal conductivity of paraffin and composite PCMs.

The thermal conductivity of the composite has a significant anisotropy, with a significant difference between radial and axial thermal conductivity. As shown in Figure 6, the maximum axial thermal conductivity of PArGO is 1.20 W/(mK) and the radial thermal conductivity is 0.54 W/(mK), compared to the pure paraffin (0.26 W/(mK)) increased by 362% and 108%, respectively. This is due to the directional arrangement of the graphene sheets in the axial direction has a larger contact area, which can reduce the contact thermal resistance and form a better thermal conductive pathway. However, the radial direction has fewer contact points and less area than the axial direction, resulting in a looser interconnection network, which increases the contact thermal resistance [31]. Because the large amount of silver particles are attached to the boundaries of the graphene sheets, the contact area with the paraffin boundaries is increased, reducing the thermal resistance of the graphene sheets to the paraffin boundaries, thus improving the thermal conductivity of the composite material. The increase of thermal conductivity of PArGO is mainly due to the addition of PrGO, which plays a key role of heat conduction network. The graphene sheets composed of PrGO pore walls have ultra-high thermal conductivity, which allow rapid heat transfer and dispersion in PArGO, increasing the area of heat transfer to paraffin, thus exhibiting faster heat transfer effect at the macroscopic level.

**Figure 7:**
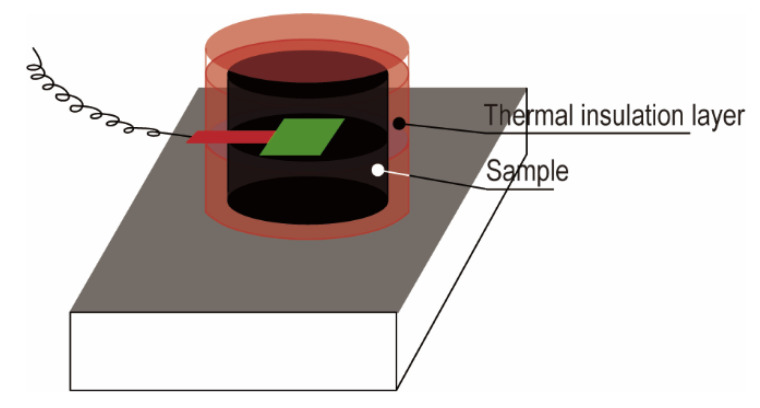
Schematic diagram simulating actual temperature changes.

**Figure 8:**
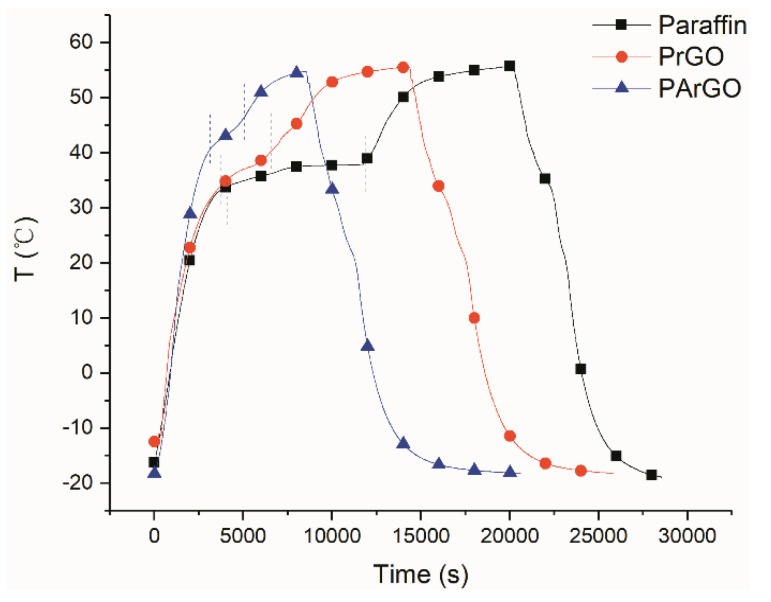
Phase transition curves of composite PCMs and paraffin in an environment simulating actual temperature changes.

The phase change behavior of the PCM was investigated in an environment simulating actual temperature changes. As in Figure 7, the sample was prepared into a cylindrical shape with the diameter of the bottom being about 4 cm and the height being about 4.5 cm. The cylindrical shape was cut in the middle and a temperature sensor was placed in it. To prevent melting leakage, the sample was encapsulated in a plastic bag and all well insulated except for the bottom. The sample is then placed on a heating plate and is subjected to programmed temperature increases and decreases to detect temperature changes in the middle of the sample. Their temperature change curves are shown in Figure 8 which can be clearly seen that the phase transition process is significantly different for different samples. The high thermal conductivity of the PArGO phase transition constant platform is narrower and the phase transition process between the two dashed lines is shorter, and the phase transition is completed in about 1990 s. The phase transition process of PrGO requires 2740 s. While paraffin has a wide phase transition platform and a significant isothermal process, which takes 7730 s to complete the phase change process. It can be seen that the addition of anisotropic graphene can improve the phase transformation efficiency of paraffin composite materials, and the thermal conductivity is further improved with silver attachment, therefore PCM can complete the phase transition much faster, increasing heat storage and release efficiency by 74%, compared with the pure paraffin.

## 4. Conclusions

In this research, anisotropic graphene aerogels were prepared by directional freezing, and then silver particles were attached on the sheet layers and boundaries of graphene by silver mirror reaction to improve the thermal conductivity of the phase change material. The samples were subjected to apparent morphology and thermal properties studies. The results show that anisotropic graphene aerogels were successfully prepared, and SEM and EDS results indicate that up to 9.14 at % silver was successfully attached to the graphene sheets and boundaries. The anisotropic thermal conductivity enhancement of the PArGO phase change composites after silver attached is significant, with a maximum axial thermal conductivity of PArGO of 1.20 W/(mK) and radial thermal conductivity of 0.54 W/(mK), compared to the pure paraffin (0.26 W/(mK)), which increased by 362% and 108% respectively. The enthalpy of the composite has been reduced to 149.6 J/g due to the silver particles attached, but the thermal performance has been greatly improved. In the experiments simulating real temperature changes, PArGO achieves phase transitions very fast, with a 74% improvement on thermal efficiency of storage and discharge over the pure paraffin. 

## Figures and Tables

**Table 1:** Content of each element in EDS face scan.

Element	Element	Intensity	Weight	Atomic
	Concentration	Calibration	Percentage	Percentage
C K ^1^	3.74	1.8581	47.68	83.59
O K ^1^	0.10	0.4336	5.52	7.27
Ag L ^1^	1.66	0.8447	46.80	9.14

^1^ C, O, and Ag respectively represent the carbon, oxygen, and silver elements. K and L respectively represent the K-shell and L-shell spectral lines of corresponding elements.
